# Development and Validation of a Machine Learning Prediction Model of Posttraumatic Stress Disorder After Military Deployment

**DOI:** 10.1001/jamanetworkopen.2023.21273

**Published:** 2023-06-30

**Authors:** Santiago Papini, Sonya B. Norman, Laura Campbell-Sills, Xiaoying Sun, Feng He, Ronald C. Kessler, Robert J. Ursano, Sonia Jain, Murray B. Stein

**Affiliations:** 1 Department of Psychiatry, University of California, San Diego, La Jolla; 2 Division of Research, Kaiser Permanente Northern California, Oakland; 3 National Center for PTSD, White River Junction, Vermont; 4 Veterans Affairs Center of Excellence for Stress and Mental Health, San Diego, California; 5 Herbert Wertheim School of Public Health and Human Longevity Science, University of California, San Diego, La Jolla; 6 Department of Health Care Policy, Harvard Medical School, Boston, Massachusetts; 7 Center for the Study of Traumatic Stress, Department of Psychiatry, Uniformed Services University of the Health Sciences, Bethesda, Maryland; 8 Psychiatry Service, Veterans Affairs San Diego Healthcare System, San Diego, California

## Abstract

**Importance:**

Military deployment involves significant risk for life-threatening experiences that can lead to posttraumatic stress disorder (PTSD). Accurate predeployment prediction of PTSD risk may facilitate the development of targeted intervention strategies to enhance resilience.

**Objective:**

To develop and validate a machine learning (ML) model to predict postdeployment PTSD.

**Design, Setting, and Participants:**

This diagnostic/prognostic study included 4771 soldiers from 3 US Army brigade combat teams who completed assessments between January 9, 2012, and May 1, 2014. Predeployment assessments occurred 1 to 2 months before deployment to Afghanistan, and follow-up assessments occurred approximately 3 and 9 months post deployment. Machine learning models to predict postdeployment PTSD were developed in the first 2 recruited cohorts using as many as 801 predeployment predictors from comprehensive self-report assessments. In the development phase, cross-validated performance metrics and predictor parsimony were considered to select an optimal model. Next, the selected model’s performance was evaluated with area under the receiver operating characteristics curve and expected calibration error in a temporally and geographically distinct cohort. Data analyses were performed from August 1 to November 30, 2022.

**Main Outcomes and Measures:**

Posttraumatic stress disorder diagnosis was assessed by clinically calibrated self-report measures. Participants were weighted in all analyses to address potential biases related to cohort selection and follow-up nonresponse.

**Results:**

This study included 4771 participants (mean [SD] age, 26.9 [6.2] years), 4440 (94.7%) of whom were men. In terms of race and ethnicity, 144 participants (2.8%) identified as American Indian or Alaska Native, 242 (4.8%) as Asian, 556 (13.3%) as Black or African American, 885 (18.3%) as Hispanic, 106 (2.1%) as Native Hawaiian or other Pacific Islander, 3474 (72.2%) as White, and 430 (8.9%) as other or unknown race or ethnicity; participants could identify as of more than 1 race or ethnicity. A total of 746 participants (15.4%) met PTSD criteria post deployment. In the development phase, models had comparable performance (log loss range, 0.372-0.375; area under the curve range, 0.75-0.76). A gradient-boosting machine with 58 core predictors was selected over an elastic net with 196 predictors and a stacked ensemble of ML models with 801 predictors. In the independent test cohort, the gradient-boosting machine had an area under the curve of 0.74 (95% CI, 0.71-0.77) and low expected calibration error of 0.032 (95% CI, 0.020-0.046). Approximately one-third of participants with the highest risk accounted for 62.4% (95% CI, 56.5%-67.9%) of the PTSD cases. Core predictors cut across 17 distinct domains: stressful experiences, social network, substance use, childhood or adolescence, unit experiences, health, injuries, irritability or anger, personality, emotional problems, resilience, treatment, anxiety, attention or concentration, family history, mood, and religion.

**Conclusions and Relevance:**

In this diagnostic/prognostic study of US Army soldiers, an ML model was developed to predict postdeployment PTSD risk with self-reported information collected before deployment. The optimal model showed good performance in a temporally and geographically distinct validation sample. These results indicate that predeployment stratification of PTSD risk is feasible and may facilitate the development of targeted prevention and early intervention strategies.

## Key Points

**Question:**

Can risk for posttraumatic stress disorder (PTSD) be accurately predicted prior to military deployment?

**Findings:**

This diagnostic/prognostic study of 4711 US Army soldiers developed machine learning models to predict postdeployment PTSD using self-reported predictors collected before deployment. An optimal model was selected based on performance in 2 cohorts and then validated in a temporally and geographically distinct cohort, where approximately one-third of participants with the highest predicted risk accounted for an estimated 62.4% of the PTSD cases.

**Meaning:**

These findings suggest that PTSD risk may be accurately stratified prior to military deployment, which may facilitate the development of targeted intervention strategies.

## Teaser

This diagnostic/prognostic study develops and validates a machine learning model to predict posttraumatic stress disorder (PTSD) diagnosis after deployment in a cohort from the Army Study to Assess Risk and Resilience in Servicemembers.

## Introduction

Posttraumatic stress disorder (PTSD) is a debilitating condition that can become chronic if untreated^1^ and is associated with psychiatric comorbidities and suicide.^2,3,4,5^ Efficient assessment of PTSD risk among those likely to experience trauma, such as soldiers, may facilitate the development of targeted preventive or early interventions that reduce individual suffering and societal costs.^6,7^ Although research has traditionally focused on isolated risk factors for PTSD,^8^ computational advances have spurred the application of machine learning (ML) to predict PTSD using individually weak predictors.^9^ Such models have the potential to facilitate research, development, and delivery of targeted intervention strategies, particularly if they rely on predictors that are easily measured.^10^ Toward this aim, we developed and validated an ML prediction model of PTSD after military deployment using predictors collected before deployment via self-report questionnaires.

Recent reviews summarize the ML literature on PTSD prediction across a range of trauma-exposed populations.^9,11,12^ However, only 2 studies^13,14^ used ML to prospectively predict PTSD among military personnel. Karstoft et al^13^ showed good prediction of PTSD (vs resilient) trajectories using predeployment self-report measures among 561 Danish soldiers deployed to Afghanistan. Schultebraucks et al^14^ showed that models exhibited a wide range of performance depending on which predictors were included among self-report, neurocognitive, and biomarker variables collected from 473 US Army soldiers deployed to Afghanistan. Machine learning was also applied to prospectively predict other mental health outcomes in soldiers, including suicide attempts,^15,16,17^ suicide deaths,^18^ depression,^19^ and psychiatric diagnosis and treatment.^20^ These studies highlight the potential of ML to quantify risk for a variety of adverse mental health outcomes that disproportionately impact soldiers.^21^

To expand on this research, we used data collected from 3 US Army brigade combat teams that deployed to Afghanistan in 2012.^22,23^ Previous research with this data set examined specific predictors of postdeployment PTSD^24,25^ or used ML to predict other psychiatric outcomes from large sets of predictors.^17,26^ Our aim was to develop and validate an ML model to predict PTSD diagnosis post deployment. In the development phase, several models that varied in their level of complexity were compared. The optimal model was then evaluated in the third cohort, which represents a rigorous test of our selected model’s temporal and geographic generalizability.

## Methods

We followed the Transparent Reporting of a Multivariable Prediction Model for Individual Prognosis or Diagnosis (TRIPOD) reporting guideline. Study procedures were approved by the ethics committees at the collaborating institutions (including Uniformed Services University of the Health Sciences for the Henry M. Jackson Foundation; Institute for Social Research at the University of Michigan, Ann Arbor; Harvard Medical School; and University of California, San Diego). Participants provided written informed consent to participate in each survey and to link their survey data and US Army and US Department of Defense administrative records.

### Study Sample

Participants were recruited from 3 cohorts of geographically distinct brigade combat teams as part of the Pre/Post Deployment Study (PPDS) of the Army Study to Assess Risk and Resilience in Servicemembers (Army STARRS).^22,23^ At predeployment, 9949 soldiers were present for duty across the 3 cohorts, of whom 7742 completed predeployment surveys and subsequently deployed to Afghanistan. Our study included the 4771 soldiers that completed 2 follow-up assessments; these participants were weighted in all analyses (ie, model development and validation). Weighting is a standard approach to address potential biases related to sample selection and follow-up nonresponse.^27,28^ A prior publication^29^ provides a detailed account of the method for estimating weights in this sample, which combines adjustment for baseline attrition, poststratification of these weights to map the sample to characteristics of the population of soldiers in the 3 brigade combat teams, and adjustment to account for loss to follow-up.

### Measures

Participants completed a self-administered computerized assessment 1 to 2 months before deployment to Afghanistan in 2012 (PPDS time 0 [T0]).^30^ Questions from the Composite International Diagnostic Interview Screening Scales^31,32^ assessed symptoms of major depression, mania and/or hypomania, panic disorder, generalized anxiety disorder, attention-deficit/hyperactivity disorder, intermittent explosive disorder, and substance use disorders. Suicidal thoughts and behavior were assessed using an expanded self-report version of the Columbia–Suicide Severity Rating Scale.^33^ Additional measures assessed childhood adversity and misconduct, lifetime trauma, 6 current and lifetime PTSD symptoms, previous deployment experiences, stress, coping styles, demographics, physical health, injuries, mental health treatment, unit experiences, weapon ownership, social networks, religiosity and/or spirituality, and personality. These measures yielded 801 potential predeployment predictors.

### Outcome

Soldiers were deployed for approximately 10 months and completed as many as 3 assessments upon return from deployment, occurring 2 to 3 weeks (PPDS T1), 2 to 3 months (PPDS T2), and 8 to 9 months (PPDS T3) post deployment. The T1 assessment included 4 PTSD symptoms and was administered within a few weeks upon return from deployment, which may be too soon to distinguish PTSD from an acute stress reaction that may subside,^34,35^ or to capture a delayed PTSD reaction.^36,37^ Therefore, we used a binary outcome defined as PTSD diagnosis (yes or no) at any point within the 2- to-9-month follow-up window that included T2 and T3. We opted for a single model to predict PTSD within this window, which was timed to rule out an acute stress response while being wide enough to capture most delayed PTSD reactions. Diagnosis was determined by survey items adapted from the PTSD Checklist–Civilian Version^38^ and Composite International Diagnostic Interview Screening Scales, which showed satisfactory concordance with independent clinical diagnosis.^31^

### Statistical Analysis

All analyses were conducted in R, version 4.2.0 (R Project for Statistical Computing).^39^ To protect against overfitting, which can lead to inflated model performance estimates, we used temporal and geographic validation: models were developed and evaluated on data from 2 cohorts (n = 3038), and the best-performing model was tested on the third cohort (n = 1733), whose predeployment data were collected in a different region of the US after the predeployment data from the other 2 cohorts.

Prior to model development, categorical features were recoded as binary indicators, and near-zero variance features were removed. For algorithms that cannot handle missing predictor data, binary missing data indicators were created, missing data were imputed with the median (for numeric features) or mode (for categorical features), and numeric features (including ordinal Likert scale responses) were standardized. To prevent information leakage, the values used for imputation and standardization were based on the development data and subsequently applied to the test data.

We considered 3 modeling strategies that varied in complexity: (1) a stacked ensemble of diverse algorithms, (2) a penalized logistic regression (elastic net) model, and (3) a gradient-boosting machine (GBM) model. The most complex model was a stacked ensemble^40,41^ of gradient boosted machines (H2O GBM and XGBoost), distributed random forests, and extremely randomized trees (hyperparameters provided in eTable 1 in Supplement 1).^42,43^ These algorithms can capture high-dimensional, nonparametric interactions among predictors and handle missingness without imputation. Predictions from these base models served as the inputs to stacked ensemble algorithms. The best-performing stacked ensemble was selected based on the lowest cross-validated log loss, which captures the difference between predicted probabilities and observed outcomes. To test whether this complex modeling strategy outperformed a simpler approach, we developed elastic net models without any interactions (logistic regression that combines least absolute shrinkage and selection operator and ridge penalties to regularize model coefficients). Elastic net models used the standardized predictor data with missing values imputed.

In addition to performance, predictor parsimony is desirable because it facilitates future model implementation by reducing the assessment burden. Elastic net can yield models with fewer predictors because regularization may reduce coefficients to zero. We also applied a novel information-theoretic approach to identify a core set of predictors that are both predictive of the outcome and not redundant with other predictors.^44^ First, we used 20% of the model development sample to identify core predictors using GBM algorithms. Core predictors were selected if they had a cross-validated normalized value of at least 0.01 on both total and net information, which reflect a predictor’s relative relevance and uniqueness, respectively. Next, we used the remaining 80% of the development sample to train up to 100 GBMs restricted to the core predictors and selected the model with the lowest cross-validated log loss.

To examine whether these models outperformed an informative benchmark (as opposed to simply prediction better than chance),^45^ we developed a univariate generalized linear model that used the predeployment score on a 6-item version of the PTSD Checklist–Civilian Version as the sole predictor. At T0, 40.1% of participants reported at least 1 PTSD symptom from prior stressful experiences.

To select an optimal model, we compared log loss and area under the receiver operating characteristics curve (AUC) estimated using 10-fold cross-validation in the development sample (8 folds for the core predictor models because the other 2 folds were used for identification of core predictors). Predictor parsimony was also considered.

Next, the optimal model was applied to the independent test data. Model discrimination was assessed with a receiver operating characteristics curve and corresponding AUC statistic. Model calibration was assessed using a logistic calibration curve and expected calibration error. Sensitivity, specificity, and positive and negative predictive values were assessed in the test sample across deciles of the predicted risk distribution from the training sample. Weighted bootstrapping (1000 replications) was used to calculate the mean of each performance metric with 95% CIs.

Additional analyses were conducted to complement our assessment of the optimal model’s overall performance. We examined the relative importance of predictors and estimated univariate associations between predictors and PTSD outcome using weighted logistic regression and reported odds ratios with SEs and *P* values. These analyses were based on the development sample because their goal is to provide potential insight into what the final model learned during the training process. We also assessed for evidence of differential model performance across age, sex, self-reported race, and ethnicity in a robust Poisson regression model of PTSD diagnosis at follow-up.^46^ In this analysis, an interaction between a sociodemographic characteristic and model-predicted probability of PTSD may indicate group differences in prediction accuracy. This analysis is based on the test sample because it is designed to assess whether implementation of the final model could potentially lead to unfair outcomes for certain groups. For example, underestimation of risk in one group may lead to reduced allocation of preventive care for that group. Two-sided *P* < .05 indicated statistical significance.

## Results

This study included 4771 participants with a mean (SD) age of 26.9 (6.2) years; 4440 (94.7%) were men and 278 (5.3%) were women. In terms of self-reported race and ethnicity, 144 (2.8%) identified as American Indian or Alaska Native, 242 (4.8%) as Asian, 556 (13.3%) as Black or African American, 885 (18.3%) as Hispanic, 106 (2.1%) as Native Hawaiian or other Pacific Islander, 3474 (72.2%) as White, and 430 (8.9%) as other or unknown race or ethnicity; participants could self-report more than 1 race or ethnicity. Additional demographic characteristics are summarized in Table 1. All analyses were weighted to address potential biases related to sample selection and missing outcome data.^29^ In the complete sample, 746 participants (15.4%) had a PTSD diagnosis at follow-up. Prevalence of PTSD was comparable across the samples used for developing models (466 [15.1%]) and testing the final model (280 [15.9%]). Performance based on lowest log loss (range, 0.372-0.375) and highest AUC (range, 0.75-0.76) was similar across the stacked ensemble, elastic net, and GBM; and all models outperformed the benchmark model (eTable 2 in Supplement 1).

**Table 1.** Participant Characteristics^a^

Characteristic	Participant group		
	Full sample (N = 4771)	Development sample (n = 3038)	Test sample (n = 1733)
Age, mean (SD), y	26.9 (6.2)	26.6 (6.1)	27.5 (6.3)
Sex			
Men	4440 (94.7)	2814 (94.3)	1626 (95.7)
Women	278 (5.3)	193 (5.7)	85 (4.3)
Spanish, Hispanic, or Latino ethnicity	885 (18.3)	581 (18.7)	304 (17.5)
Race^b^			
American Indian or Alaska Native	144 (2.8)	90 (2.8)	54 (2.8)
Asian	242 (4.8)	140 (4.4)	102 (5.5)
Black or African American	556 (13.3)	343 (12.3)	213 (15.0)
Native Hawaiian or other Pacific Islander	106 (2.1)	54 (1.7)	52 (3.0)
White	3474 (72.2)	2248 (73.1)	1226 (70.4)
Other or unknown^c^	430 (8.9)	264 (8.5)	166 (9.7)
Married	2638 (57.4)	1609 (54.9)	1029 (62.3)
Highest level of education completed			
GED or equivalent	294 (6.4)	171 (5.8)	123 (7.6)
High school diploma	1824 (36.3)	1150 (36.1)	674 (36.6)
Post–high school education but no degree	1314 (26.8)	831 (26.8)	483 (26.7)
Technical school certificate or degree	303 (5.7)	201 (6.1)	102 (5.1)
2-y College degree	372 (7.9)	235 (7.7)	137 (8.3)
4-y College degree	529 (13.3)	360 (13.8)	169 (12.3)
Graduate or professional study	110 (3.5)	74 (3.7)	36 (3.3)

Abbreviation: GED, General Educational Development.

^a^ The number of participants with missing data was 141 for age (81 in the development sample and 60 in the test sample); 53 for sex (31 in the development sample and 22 in the test sample); 26 for marital status (18 in the development sample and 8 in the test sample); and 25 for educational attainment (16 in the development sample and 9 in the test sample). Unless otherwise indicated, data are expressed as No. (weighted %) of participants.

^b^ Participants had the option of self-reporting more than 1 race.

^c^ Participants endorsed the category other or did not endorse any of the available categories.

**Figure 1.**
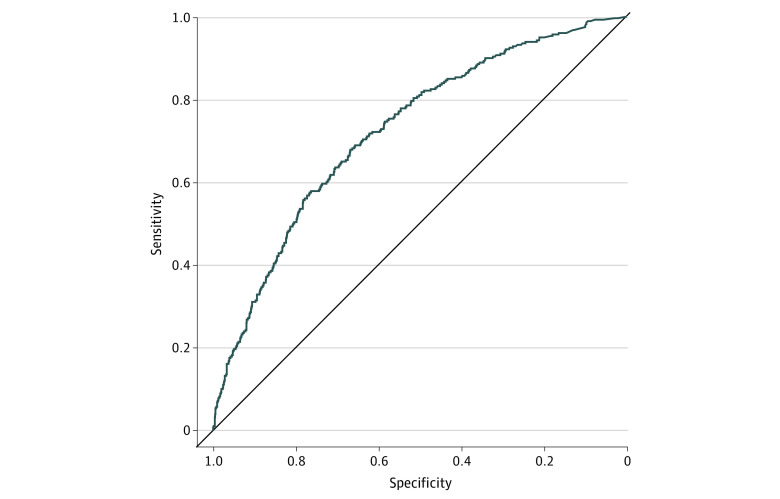
Receiver Operating Characteristics Curve Based on the Core Predictor Gradient-Boosting Machine Model’s Predictions of Postdeployment Posttraumatic Stress Disorder in the Test Sample (n = 1733)

**Figure 2.**
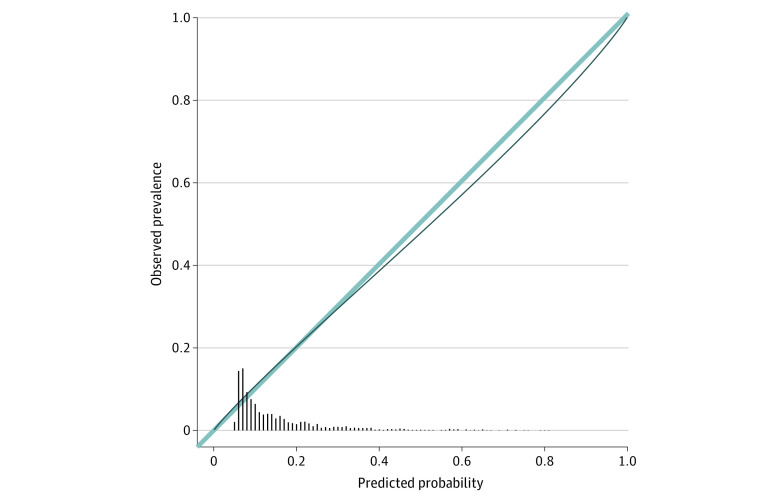
Logistic Calibration Curve Based on the Core Predictor Gradient-Boosting Machine Model’s Predictions of Postdeployment Posttraumatic Stress Disorder in the Test Sample (n = 1733)

We selected the GBM with 58 core predictors because it achieved comparable performance to the stacked ensemble with 801 predictors and elastic net with 196 predictors. When applied to the independent test sample, the core predictor GBM showed good discrimination (AUC = 0.74 [95% CI, 0.71-0.77]) (Figure 1), and good calibration (expected calibration error, 0.032 [95% CI, 0.020-0.046]) (Figure 2). Table 2 contains threshold-dependent performance metrics of the core predictor GBM model in the test sample across deciles of the predicted risk distribution based on thresholds from the training sample. Approximately one-third of participants in the test sample (33.9% [95% CI, 31.7%-36.1%]) had predicted probabilities in the top 3 risk deciles; these participants accounted for 62.4% (95% CI, 56.5%-67.9%) of PTSD cases.

**Table 2.** Performance Metrics of the Core Predictor GBM Model in the Test Sample Across Deciles of the Predicted Risk Distribution Based on Thresholds From the Training Sample

Risk decile	% (95% CI)		Mean (95% CI)			
	Participants within decile	Cumulative participants	Cumulative sensitivity	Cumulative specificity	Cumulative PPV	Cumulative NPV
1	11.6 (10.1-13.2)	11.6 (10.1-13.2)	28.6 (23.2-34.3)	91.7 (90.2-93.1)	39.5 (32.4-46.6)	87.1 (85.4-88.8)
2	11.5 (10.1-13.1)	23.1 (21.1-25.0)	49.6 (43.5-55.2)	81.9 (79.8-83.8)	34.3 (29.8-39.0)	89.6 (87.9-91.1)
3	10.8 (9.4-12.2)	33.9 (31.7-36.1)	62.4 (56.5-67.9)	71.5 (69.2-73.7)	29.4 (25.8-33.0)	90.9 (89.3-92.5)
4	11.0 (9.6-12.5)	44.9 (42.5-47.1)	74.0 (68.6-79.0)	60.7 (58.2-63.0)	26.3 (23.2-29.4)	92.5 (90.8-94.1)
5	9.5 (8.1-11.0)	54.4 (52.0-56.6)	82.1 (77.9-86.6)	50.9 (48.5-53.4)	24.1 (21.5-26.9)	93.8 (92.1-95.4)
6	10.9 (9.5-12.4)	65.3 (63.2-67.4)	88.2 (84.5-92.0)	39.1 (36.7-41.4)	21.6 (19.3-24.1)	94.6 (92.7-96.3)
7	10.2 (8.8-11.5)	75.4 (73.5-77.3)	94.2 (91.4-96.9)	28.1 (25.9-30.1)	19.9 (17.8-22.1)	96.2 (94.4-97.9)
8	9.2 (8.0-10.6)	84.7 (83.0-86.3)	96.8 (94.8-98.6)	17.7 (15.8-19.6)	18.2 (16.3-20.3)	96.7 (94.7-98.6)
9	10.3 (8.9-11.8)	95.0 (93.9-95.9)	99.5 (98.5-100)	5.9 (4.8-7.1)	16.7 (14.9-18.5)	98.4 (95.4-100)
10	5.0 (4.1-6.1)	100	100	0	16.0 (14.2-17.7)	0

Abbreviations: GBM, gradient-boosting machine; NPV, negative predictive value; PPV, positive predictive value.

eTables 3 and 4 in Supplement 1 provide information about the 58 core predictors, including the normalized estimates of total information and net information (higher means more predictive of the outcome and more unique information contributed to the prediction, respectively). Given that a key criterion of core predictors is that they contain unique predictive information, it is not surprising that core predictors cut across 17 distinct domains in the predeployment assessment, including stressful experiences, social network, substance use, childhood or adolescence, unit experiences, health, injuries, irritability or anger, personality, emotional problems, resilience, treatment, anxiety, attention or concentration, family history, mood, and religion. Moreover, some of the core predictors with the lowest total predictive information had the highest net predictive information (ie, unique predictive information given all other predictors). This reflects the selection of predictors that can fill in predictive gaps because they do not overlap with other stronger predictors.

**Figure 3.**
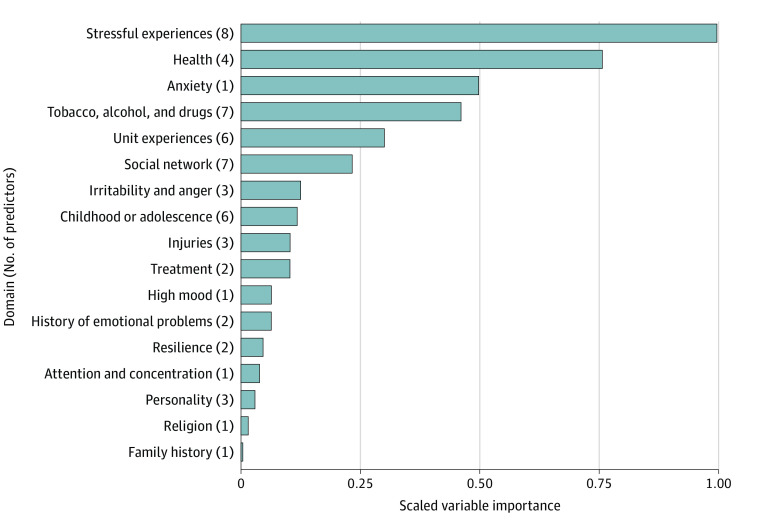
Scaled Predictor Importance Grouped by Domain in the Core Predictor Gradient-Boosting Machine Model

The individual contribution of each core predictor to the final model is captured by its scaled importance (eTable 3 in Supplement 1). Figure 3 illustrates the importance of predictors grouped by domain. In the univariate logistic regression analyses that examined linear associations between each predictor and PTSD at follow-up, odds ratios ranged from 0.69 to 2.46. Without taking statistical significance into account, 72.4% of predictors were associated with greater odds and 27.6% with lower odds of PTSD at follow-up. Half of the core predictors (n = 29) did not have a univariate association with the target outcome at the Bonferroni-corrected threshold (*P* > .05/58), which highlights the GBM algorithm’s capacity to identify variables whose predictive utility comes from nonlinear and/or interactive associations with PTSD diagnosis at follow-up. While these analyses provide useful insight into the information that was used by the final model to make its predictions, they are not designed to support causal interpretations.

eTable 5 in Supplement 1 contains results from the Poisson regression that assessed for potential differences in the association between the optimal GBM’s model-predicted PTSD probabilities and actual PTSD outcomes across sociodemographic groups. There were no significant interactions between the selected model’s predictions and the self-reported characteristics of age, sex, ethnicity, or race (*P* >.17). This suggests that the association between the GBM model’s predictions and the observed outcomes was not significantly different across sociodemographic groups.

## Discussion

We developed models to predict PTSD 2 to 9 months post deployment using self-reported data collected before deployment from 2 US Army brigade combat teams and validated the optimal model in a third temporally and geographically distinct cohort. In the development phase, all models outperformed a benchmark univariate generalized linear model. A GBM model that used only 58 core predictors was selected as the optimal model because it achieved comparable performance to the alternative models, despite relying only on approximately 7% of the available predictors.

The optimal model had a similar AUC when it was applied to the independent test sample, indicating good temporal and geographic generalization. Several differences should be noted when comparing our model’s AUC of 0.74 (95% CI, 0.71-0.77) with the best-performing models developed in other samples of military personnel deployed to Afghanistan.^13,14^ For example, the best-performing models of Schultebraucks et al^14^ had biomarkers and neurocognitive predictors in addition to self-report; in models that were restricted to self-report predictors only, the AUC ranged from 0.63 to 0.69 depending on the modeling strategy. Karstoft et al^13^ found relatively better performance with self-reported predictors (AUC = 0.84 [95% CI, 0.81-0.87]) using repeated cross-validation such that all participants (N = 561) were iteratively used in the development and validation of their models. We validated models on a temporally and geographically distinct data set, which is arguably a more rigorous test of model generalizability compared with the use of random folds in the prior studies. We also used weighting to adjust the sample so that it more closely resembled the target population, which reduces the potential for biases arising from sample selection and missing outcome data. Finally, our considerably larger sample, which included 280 PTSD cases in our test data set compared with 9 PTSD cases reported by Schultebraucks et al,^14^ enhanced the precision of our performance estimates. Altogether, this work shows that ML models can achieve good prediction of postdeployment PTSD using a wide range of methods and predictors, across different samples and research teams.

A key innovation of our study is the use of a novel information-theoretic approach to identifying strong and unique predictors.^44^ Only half of these had univariate associations with our target outcome. Thus, a benefit of GBMs is that strong univariate predictors are identified alongside predictors that may have nonlinear associations with the outcome and may interact with other predictors to increase model performance. While these analyses should not be causally interpreted, they aid in the interpretability of our model. Moreover, our selection of the model that relies on 58 core predictors represents over 90% reduction in the number of necessary predictors compared with the full model, which can facilitate model implementation by reducing assessment burden.

### Limitations

This study has some limitations. The self-report questionnaires may have yielded socially desirable or otherwise biased responses from some participants. The predeployment assessments cut across multiple domains; however, other information that can be collected via self-report likely has additional predictive value. Although we used temporal and geographic validation in the context of data from Operation Enduring Freedom, additional external validation is required to further assess the transportability of the model to other components of the US Army or to soldiers deployed to other locations and at different times. Nevertheless, our model provides valuable information about which items may be most informative for predicting PTSD among soldiers who may be involved in future combat situations. Finally, there is growing recognition of the need to assess model fairness, and many criteria (some mutually exclusive) have been proposed.^47,48^ There was no significant evidence of differential associations between model predictions and postdeployment PTSD outcomes across sociodemographic groups. However, some subgroups were small (eg, women, American Indian or Alaska Native individuals, Native Hawaiian or other Pacific Islander individuals); therefore, the lack of significant differences should not be interpreted as direct evidence of model fairness. Although analyses were weighted to reduce the potential for selection and attrition biases, the presence of unmeasured confounding is a possibility. Finally, it is important to note that feasibility of model implementation hinges on additional factors that were beyond the scope of the study, including cost-effectiveness research that takes multiple intervention parameters into account to determine model accuracy requirements, and thorough evaluation of ethical implications so that model implementation does not inadvertently lead to unfair outcomes. For example, using the model for targeting predeployment resilience training would have different costs, benefits, and ethical implications than for targeting enhanced PTSD screening post deployment.

## Conclusions

In this diagnostic/prognostic study of US Army soldiers, an ML model was developed to predict postdeployment PTSD risk with self-reported information collected before deployment. The optimal model showed good performance in a temporally and geographically distinct validation sample. Although these results suggest that it may be possible to stratify PTSD risk in similar scenarios, further work is necessary to determine appropriate thresholds for targeted intervention, which requires careful evaluation of cost-effectiveness and ethical implications.

## Notes

Supplement 1. **eTable 1.** Random Grid Search Parameters Used for Tuning Each Algorithm **eTable 2.** Metrics of the Best-Performing Model for Each Strategy Used in the Development Phase **eTable 3.** Core Predictor Importance Statistics Arranged in Order of Scaled Variable Importance **eTable 4.** Descriptive Statistics of the Core Predictors **eTable 5.** Associations Between Model-Predicted Probabilities and Observed Outcomes as a Function of Age, Sex, Ethnicity, and Race in the Test Sample

Supplement 2.
Data Sharing Statement
