# Supplementary material for: Development and Validation of a Machine Learning Prediction Model of Posttraumatic Stress Disorder After Military Deployment
Source: JAMA Netw Open. 2023 Jun 30;6(6):e2321273. doi: 10.1001/jamanetworkopen.2023.21273 (PMC10314304; doi:10.1001/jamanetworkopen.2023.21273)
Supplement: Supplement 1. — eTable 1. Random Grid Search Parameters Used for Tuning Each Algorithm eTable 2. Metrics of the Best-Performing Model for Each Strategy Used in the Development Phase eTable 3. Core Predictor Importance Statistics Arranged in Order of Scaled Variable Importance eTable 4. Descriptive Statistics of the Core Predictors eTable 5. Associations Between Model-Predicted Probabilities and Observed Outcomes as a Function of Age, Sex, Ethnicity, and Race in the Test Sample [file jamanetwopen-e2321273-s001.pdf]

Supplementary Online Content

Papini S, Norman SB, Campbell-Sills L, et al. Development and validation of a machine learning prediction model of posttraumatic stress disorder after military deployment. *JAMA Netw Open*. 2023;6(6):e2321273. doi:10.1001/jamanetworkopen.2023.21273

eTable 1. Random Grid Search Parameters Used for Tuning Each Algorithm
eTable 2. Metrics of the Best-Performing Model for Each Strategy Used in the Development Phase
eTable 3. Core Predictor Importance Statistics Arranged in Order of Scaled Variable Importance
eTable 4. Descriptive Statistics of the Core Predictors
eTable 5. Associations Between Model-Predicted Probabilities and Observed Outcomes as a Function of Age, Sex, Ethnicity, and Race in the Test Sample

This supplementary material has been provided by the authors to give readers additional information about their work.

© 2023 Papini S et al. *JAMA Network Open*.

eTable 1. Random Grid Search Parameters Used for Tuning Each Algorithm

Algorithm	Parameter	Search Values
Elastic net	lambda	h2o default
	alpha	0, 0.1, 0.2, 0.3, 0.4, 0.5, 0.6, 0.7, 0.8, 0.9, 1
XGBoost	booster	gbtree, dart
	col_sample_rate	0.6, 0.8, 1.0
	col_sample_rate_per_tree	0.7, 0.8, 0.9, 1.0
	max_depth	5, 10, 15, 20
	min_rows	0.01, 0.1, 1.0, 3.0, 5.0, 10.0, 15.0, 20.0
	ntrees	10000
	reg_alpha	0.001, 0.01, 0.1, 1, 10, 100
	reg_lambda	0.001, 0.01, 0.1, 0.5, 1
	sample_rate	0.6, 0.8, 1.0
Gradient boosting machine (GBM)	col_sample_rate	0.4, 0.7, 1.0
	col_sample_rate_per_tree	0.4, 0.7, 1.0
	learn_rate	0.1
	max_depth	3, 4, 5, 6, 7, 8, 9, 10, 11, 12, 13, 14, 15, 16, 17
	min_rows	1, 5, 10, 15, 30, 100
	min_split_improvement	1e-4, 1e-5
	ntrees	10000
	sample_rate	0.50, 0.60, 0.70, 0.80, 0.90, 1.00
Distributed random forest (DFR) and Extremely randomized trees (XRT)	col_sample_rate_change_per_level	1
	col_sample_rate_per_tree	1
	histogram_type	AUTO (DRF); Random (XRT)
	max_depth	20
	min_rows	1
	min_split_improvement	1e-05
	mtries	sqrt(number of predictors)
	nbins	20
	ntrees	50
	sample_rate	0.6320000291

Note. Nested cross-validation is used to select the optimal hyperparameters for the elastic net, XGBoost, and GBM algorithms. Only one DRF and one XRT model is trained using the default parameters listed above. Additional documentation is available at https://docs.h2o.ai/h2o/latest-stable/h2o-docs

© 2023 Papini S et al. *JAMA Network Open*.

eTable 2. Metrics of the Best-Performing Model for Each Strategy Used in the Development Phase

Modelling strategy	Number of predictors	Cross-validated log-loss	Cross-validated AUC
stacked ensemble	801	0.372	0.76
elastic net	196	0.374	0.75
gradient boosting machine with core predictors	58	0.375	0.75
benchmark logistic regression	1	0.391	0.67

Note. Log-loss reflects the difference between predicted probabilities and observed outcomes (lower is better); Area under the receiver operating characteristics curve (AUC) reflects the trade-off between sensitivity and specificity (higher is better).

© 2023 Papini S et al. *JAMA Network Open*.

eTable 3. Core Predictor Importance Statistics Arranged in Order of Scaled Variable Importance

Predictor description	Category	Total info.	Net info.	Scaled importance	Odds Ratio	SE	P-value
feeling restless, fidgety, keyed up (frequency, past month)	Anxiety	0.05	0.04	1.00	1.30	1.1	.008
sleep problems (frequency, past month)	Health	0.01	0.03	0.97	2.01	1.05	< .001
feeling jumpy or easily startled (severity, past month)	Stressful experiences	0.05	0.03	0.70	1.73	1.04	< .001
emotionally numb after stressful experience (severity, worst month)	Stressful experiences	0.05	0.26	0.59	1.70	1.05	< .001
expected pain (severity, future 5 years)	Health	0.07	0.28	0.48	1.55	1.07	< .001
first use of 5 or more alcoholic drinks (age)	Tobacco, alcohol, & drugs	0.02	0.16	0.24	0.98	1.05	.673
age at first alcohol or drug problem	Tobacco, alcohol, & drugs	0.03	0.10	0.23	1.28	1.08	.002
difficulty concentrating after stressful experience (severity, past month)	Stressful experiences	0.04	0.06	0.20	1.56	1.04	< .001
feeling jumpy or easily startled (severity, worst month)	Stressful experiences	0.45	0.19	0.19	1.71	1.05	< .001
stopped counseling and talked to friends/family instead	Treatment	0.04	0.04	0.17	0.99	1.13	.933
unit leaders embarrass soldiers (frequency)	Unit experiences	0.25	0.57	0.17	1.40	1.05	< .001
unit leaders show concern for safety (frequency)	Unit experiences	0.04	0.22	0.16	0.84	1.05	< .001
age when first had nicotine dependence	Tobacco, alcohol, & drugs	0.01	0.05	0.16	0.91	1.1	.326
likely to seek help from mental health counselor if needed	Social network	0.14	0.23	0.15	1.12	1.05	.031
feeling discriminated against because of age, gender, race, or ethnicity	Unit experiences	0.03	0.09	0.14	1.27	1.04	< .001
explosive anger (frequency)	Irritability & anger	1.00	1.00	0.14	1.60	1.04	< .001

© 2023 Papini S et al. *JAMA Network Open*.

Predictor description	Category	Total info.	Net info.	Scaled importance	Odds Ratio	SE	P-value
injury-related memory lapse that lasted less than 30 mins (frequency, lifetime)	Injuries	0.01	0.02	0.13	1.23	1.08	.011
nicotine dependence (months, past year)	Tobacco, alcohol, & drugs	0.07	0.04	0.13	1.23	1.09	.014
episode when talked so much others could not (frequency)	High mood	0.10	0.06	0.13	1.35	1.08	< .001
alcohol use (frequency, past month)	Tobacco, alcohol, & drugs	0.02	0.07	0.11	1.07	1.05	.201
career-related stress (severity, past year)	Stressful experiences	0.12	0.17	0.11	1.56	1.05	< .001
days unavailable in unit (frequency, past 3 months)	Unit experiences	0.05	0.34	0.10	0.97	1.05	.584
number of biological children less than 6 years old	Social network	0.03	0.13	0.09	1.04	1.05	.473
steal from others (frequency, before age 18)	Childhood & adolescence	0.01	0.16	0.09	1.32	1.04	< .001
reactions to stress interfered with work or personal life (frequency, past month)	Stressful experiences	0.05	0.08	0.09	1.47	1.04	< .001
anger attacks interfered with personal life (frequency)	Irritability & anger	0.07	0.05	0.09	1.49	1.08	< .001
comfortable with others or alone, not concerned with rejection	Social network	0.02	0.08	0.08	0.90	1.05	.035
age at first knock-out injury	Injuries	0.10	0.28	0.08	1.27	1.08	.002
fast or unsafe driving (frequency, past 6 months)	Attention and Concentration	0.01	0.11	0.08	1.31	1.09	.001
difficulty remembering stressful experience (severity, worst month)	Stressful experiences	0.02	0.03	0.08	1.54	1.04	< .001
number of people that rely on participant for help	Social network	0.07	0.11	0.07	0.90	1.05	.044
age when first shy or scared of social situations	History of emotional problems	0.02	0.12	0.07	1.34	1.09	.001
likely to rely on religious counselor if needed	Social network	0.10	0.06	0.06	0.92	1.05	.090

© 2023 Papini S et al. *JAMA Network Open*.

Predictor description	Category	Total info.	Net info.	Scaled importance	Odds Ratio	SE	P-value
shy or scared in social situations (frequency, lifetime)	History of emotional problems	0.01	0.07	0.06	0.93	1.1	.456
energy pills used (quantity, past month)	Tobacco, alcohol, & drugs	0.05	0.31	0.06	1.10	1.05	.028
stealing, shoplifting, forging signatures before age 18 (frequency)	Childhood & adolescence	0.02	0.03	0.06	1.25	1.04	< .001
years lived with biological mother (before age 18)	Childhood & adolescence	0.03	0.15	0.05	0.87	1.05	.004
has difficulty adjusting to changes	Resilience	0.04	0.35	0.05	1.37	1.05	< .001
health-related interference with relationships (severity, past month)	Health	0.11	0.42	0.05	1.61	1.04	< .001
current morale in unit (very low to very high)	Unit experiences	0.04	0.11	0.04	0.69	1.05	< .001
ability to maintain sense of humor in tense situations	Resilience	0.03	0.19	0.04	0.84	1.05	.001
self-conscious	Personality	0.05	0.26	0.04	1.11	1.05	.032
level of spirituality	Religion	0.03	0.01	0.03	1.01	1.05	.838
did mean things as payback (frequency, before age 18)	Childhood & adolescence	0.03	0.22	0.03	1.33	1.05	< .001
did not get promoted (yes/no, past year)	Stressful experiences	0.12	0.34	0.03	2.19	1.14	< .001
feeling a need to receive counseling (yes/no, past year)	Treatment	0.03	0.07	0.03	2.46	1.23	< .001
resentful	Personality	0.02	0.04	0.02	1.20	1.05	< .001
feeling irritated (frequency)	Irritability & anger	0.01	0.05	0.02	1.56	1.05	< .001
number of activity partners	Social network	0.03	0.11	0.01	0.80	1.05	< .001
mother was afraid of specific things (yes/no)	Family history	0.03	0.21	0.01	1.34	1.18	.083
muscle tension (frequency, past month)	Health	0.03	0.27	0.01	1.52	1.05	< .001
leaders interested in well-being of soldiers	Unit experiences	0.04	0.10	0	0.81	1.05	< .001

© 2023 Papini S et al. *JAMA Network Open*.

Predictor description	Category	Total info.	Net info.	Scaled importance	Odds Ratio	SE	P-value
length of current romantic relationship	Social network	0.05	0.02	0	0.86	1.13	.232
age when parents divorced	Childhood & adolescence	0.17	0.24	0	0.90	1.08	.164
modest, does not brag about accomplishments	Personality	0.10	0.09	0	1.05	1.05	.360
number of younger siblings	Childhood & adolescence	0.09	0.17	0	1.09	1.05	.073
consumed 5 or more alcoholic drinks (frequency, past month)	Tobacco, alcohol, & drugs	0.03	0.03	0	1.14	1.05	.011
dazed and confused after injury (frequency, lifetime)	Injuries	0.05	0.08	0	1.24	1.05	< .001

Note. Core predictors were selected if they had a total- and net-information value of at least 0.01 (normalized on 0-1 range) in 20% of the development sample. Scaled importance is based on the core-predictor GBM model trained on 80% of the development sample. Odds ratios (OR) with standard errors (SE) and uncorrected *P*-values are based on weighted univariate logistic regressions examining the association between each predictor (standardized) and PTSD status at 3-month follow-up in the complete development sample.

© 2023 Papini S et al. *JAMA Network Open*.

eTable 4. Descriptive Statistics of the Core Predictors

	Model Development Set *N* = 3038			Model Testing Set *N* = 1733		
Predictor description	Range	Mean (SD) or % yes	% missing	Range	Mean (SD) or % yes	% missing
feeling restless, fidgety, keyed up (frequency, past month)	1-5	3.05 (1.21)	85	1-5	3.04 (1.12)	83
sleep problems (frequency, past month)	1-5	2.52 (1.35)	0	1-5	2.64 (1.35)	0
feeling jumpy or easily startled (severity, past month)	1-5	1.41 (0.84)	15	1-5	1.44 (0.87)	15
emotionally numb after stressful experience (severity, worst month)	1-5	1.55 (1.03)	15	1-5	1.62 (1.13)	16
expected pain (severity, future 5 years)	0-10	4.44 (2.76)	56	0-10	4.64 (2.74)	49
first use of 5 or more alcoholic drinks (age)	1-23	4.6 (2.8)	0	1-32	4.76 (2.99)	1
age at first alcohol or drug problem	1-23	6.75 (3.79)	74	1-31	6.8 (3.97)	73
difficulty concentrating after stressful experience (severity, past month)	1-5	1.34 (0.78)	15	1-5	1.46 (0.92)	15
feeling jumpy or easily startled (severity, worst month)	1-5	1.56 (0.99)	16	1-5	1.61 (1.05)	16
stopped counseling and talked to friends/family instead	1-4	2.03 (1.13)	88	1-4	2.11 (1.18)	91
unit leaders embarrass soldiers (frequency)	1-5	2.58 (1.18)	1	1-5	2.65 (1.19)	0
age when first had nicotine dependence	1-30	7.12 (4.37)	75	1-25	6.61 (4.27)	74
unit leaders show concern for safety (frequency)	1-5	4.03 (1.06)	1	1-5	3.85 (1.08)	0
likely to seek help from mental health counselor if needed	1-5	2.63 (1.38)	1	1-5	2.65 (1.35)	1
explosive anger (frequency)	1-5	1.64 (0.93)	0	1-5	1.67 (0.98)	0
feeling discriminated against because of age, gender, race, or ethnicity	1-4	1.28 (0.7)	1	1-4	1.33 (0.74)	0

© 2023 Papini S et al. *JAMA Network Open*.

	Model Development Set N = 3038			Model Testing Set N = 1733		
Predictor description	Range	Mean (SD) or % yes	% missing	Range	Mean (SD) or % yes	% missing
episode when talked so much others could not (frequency)	1-5	1.79 (1.01)	75	1-5	1.81 (1.06)	75
injury-related memory lapse that lasted less than 30 mins (frequency, lifetime)	0-10	1.21 (1.71)	77	0-10	1.27 (1.92)	79
nicotine dependence (months, past year)	0-9	2.77 (2.99)	75	0-9	3.01 (3.02)	74
alcohol use (frequency, past month)	1-5	2.51 (1.07)	6	1-5	2.5 (1.13)	7
career-related stress (severity, past year)	1-5	1.93 (1.01)	1	1-5	1.96 (1.05)	0
days unavailable in unit (frequency, past 3 months)	0-8	2.68 (2.87)	2	0-8	2.53 (2.75)	1
anger attacks interfered with personal life (frequency)	1-5	2.11 (1.14)	69	1-5	2.06 (1.13)	71
number of biological children less than 6 years old	0-5	0.44 (0.73)	3	0-5	0.53 (0.8)	4
reactions to stress interfered with work or personal life (frequency, past month)	1-5	1.15 (0.54)	15	1-5	1.19 (0.58)	15
steal from others (frequency, before age 18)	1-5	1.06 (0.36)	2	1-5	1.05 (0.31)	2
age at first knock-out injury	1-23	3.8 (4.03)	73	1-24	3.59 (4.03)	74
comfortable with others or alone, not concerned with rejection	1-5	3.36 (1.35)	1	1-5	3.25 (1.41)	1
difficulty remembering stressful experience (severity, worst month)	1-5	1.35 (0.81)	15	1-5	1.38 (0.88)	16
fast or unsafe driving (frequency, past 6 months)	1-5	2.48 (1.32)	74	1-5	2.27 (1.26)	71
age when first shy or scared of social situations	1-30	3.42 (4.13)	80	1-27	3.18 (4.08)	76
number of people that rely on participant for help	0-9	4.31 (2.32)	1	0-9	4.03 (2.28)	1
energy pills used (quantity, past month)	1-7	1.14 (0.73)	23	1-7	1.17 (0.78)	26

© 2023 Papini S et al. *JAMA Network Open*.

		Model Development Set<br>*N* = 3038			Model Testing Set<br>*N* = 1733	
Predictor description	Range	Mean (SD) or % yes	% missing	Range	Mean (SD) or % yes	% missing
likely to rely on religious counselor if needed	1-5	2.98 (1.38)	1	1-5	2.85 (1.35)	1
shy or scared in social situations (frequency, lifetime)	1-10	5.03 (3.36)	80	1-10	5.27 (3.5)	76
stealing, shoplifting, forging signatures before age 18 (frequency)	1-5	1.32 (0.71)	2	1-5	1.29 (0.64)	2
has difficulty adjusting to changes	1-5	1.66 (0.92)	2	1-5	1.74 (0.98)	1
health-related interference with relationships (severity, past month)	0-10	1.6 (2.51)	0	0-10	1.67 (2.51)	0
years lived with biological mother (before age 18)	0-8	7.14 (2.06)	2	0-8	7.25 (1.89)	1
ability to maintain sense of humor in tense situations	1-5	4.2 (0.95)	1	1-5	4.14 (0.99)	0
current morale in unit (very low to very high)	1-5	3.52 (1.05)	1	1-5	3.36 (1.03)	1
self-conscious	1-5	2.43 (1.27)	2	1-5	2.55 (1.29)	1
did mean things as payback (frequency, before age 18)	1-5	1.45 (0.85)	3	1-5	1.48 (0.9)	2
did not get promoted (past year)	yes/no	13.5	1	yes/no	13.9	0
feeling a need to receive counseling (past year)	yes/no	5.9	18	yes/no	6.7	16
level of spirituality	1-4	2.56 (1.03)	1	1-4	2.51 (1.05)	1
feeling irritated (frequency)	1-5	2.3 (1.01)	0	1-5	2.35 (1.02)	0
resentful	1-5	1.7 (0.91)	1	1-5	1.75 (0.95)	1
mother was afraid of specific things	yes/no	9.1	3	yes/no	9.6	3
muscle tension (frequency, past month)	1-5	1.96 (1.06)	0	1-5	2.08 (1.11)	0
number of activity partners	0-9	4.01 (2.32)	1	0-9	3.64 (2.24)	0
age when parents divorced	1-5	1.93 (1.3)	55	1-5	1.95 (1.3)	53
consumed 5 or more alcoholic drinks (frequency, past month)	1-5	1.9 (0.96)	6	1-5	1.83 (0.95)	7

© 2023 Papini S et al. *JAMA Network Open*.

	Model Development Set<br>N = 3038			Model Testing Set<br>N = 1733		
Predictor description	Range	Mean (SD) or % yes	% missing	Range	Mean (SD) or % yes	% missing
dazed and confused after injury (frequency, lifetime)	0-10	2.21 (2.83)	0	0-10	2.23 (2.88)	1
leaders interested in well-being of soldiers	1-5	3.81 (1.16)	1	1-5	3.58 (1.18)	0
length of current romantic relationship	1-7	2.33 (1.34)	81	1-7	2.33 (1.39)	84
modest, does not brag about accomplishments	1-5	3.33 (1.17)	1	1-5	3.26 (1.2)	1
number of younger siblings	0-6	1.34 (1.38)	2	0-6	1.34 (1.41)	2

Note. To facilitate the interpretation of descriptive statistics, all Likert scale responses have all been re-coded such that greater values reflect higher levels, frequency, or severity of the predictor. Means (M), standard deviations (SD), and proportions (%; for binary predictors) reflect weighted statistics. Note that the optimal model was a Gradient Boosting Machine (GBM), which handles missingness by creating a split in the decision tree that captures whether or not a predictor has missing data. This approach treats missingness as an informative category (as opposed to assuming values are missing at random).

© 2023 Papini S et al. *JAMA Network Open*.

eTable 5. Associations Between Model-Predicted Probabilities and Observed Outcomes as a Function of Age, Sex, Ethnicity, and Race in the Test Sample

	Relative Risk	Standard Error	*P-value*
Model-predicted probability of PTSD (PP)	22.23	1.13	.01
Age	1.00	0.01	.82
Female sex	0.73	0.42	.44
Spanish/Hispanic/Latino Ethnicity	2.04	0.26	.01
Asian	1.17	0.48	.74
White	1.01	0.37	.98
Black or African American	0.87	0.40	.73
American Indian or Alaskan Native	1.49	0.41	.33
Native Hawaiian or other Pacific Islander	1.35	0.60	.62
Other or unrecorded race	0.75	0.48	.54
PP × age	1.03	0.04	.41
PP × female sex	0.99	1.10	.99
PP × Spanish/Hispanic/Latino Ethnicity	0.36	0.92	.27
PP × American Indian or Alaskan Native	0.86	0.84	.85
PP × Asian	0.33	1.76	.53
PP × Black or African American	1.61	0.84	.57
PP × Native Hawaiian or other Pacific Islander	0.20	1.20	.17
PP × Other or unrecorded race	1.48	1.33	.77
PP × White	0.69	0.80	.64

Note. All interactions between model-predicted probability of PTSD and sociodemographic characteristic were non-significant (all *P*s > .17).

© 2023 Papini S et al. *JAMA Network Open*.
