# Supplementary material for: Development and Validation of a Machine Learning Prediction Model of Posttraumatic Stress Disorder After Military Deployment
Source: JAMA Netw Open. 2023 Jun 30;6(6):e2321273. doi: 10.1001/jamanetworkopen.2023.21273 (PMC10314304; doi:10.1001/jamanetworkopen.2023.21273)
Supplement: Supplement 2. — Data Sharing Statement [file jamanetwopen-e2321273-s002.pdf]

Data Sharing Statement

Papini. Development and Validation of a Machine Learning Prediction Model of Posttraumatic Stress Disorder After Military Deployment. *JAMA Netw Open*. Published June 30, 2023. doi:10.1001/jamanetworkopen.2023.21273

Data
Data available: No

Additional Information
Explanation for why data not available: Data for STARRS surveys are available through the University of Michigan's Inter-university Consortium for Political and Social Research (ICPSR) to researchers who have IRB approval.
